# Associations of Place-Based Factors with Service Use and Consumer-Reported Unmet Service Needs Among Older Adults Using Publicly Funded Home- and Community-Based Services in the United States

**DOI:** 10.3390/ijerph22091461

**Published:** 2025-09-22

**Authors:** Tetyana P. Shippee, Romil R. Parikh, Nicholas Musinguzi, Benjamin W. Langworthy, Jack M. Wolf, Stephanie Giordano, Eric Jutkowitz

**Affiliations:** 1 Division of Health Policy and Management, School of Public Health, University of Minnesota, Minneapolis, MN 55455, USA; 2 Division of Biostatistics and Health Data Science, School of Public Health, University of Minnesota, Minneapolis, MN 55414, USA; 3 Human Services Research Institute, Cambridge, MA 02140, USA

**Keywords:** neighborhood, environment, social determinants of health, long-term services and supports, aging in place, home healthcare

## Abstract

Access to home- and community-based services (HCBS) is critical for aging in place; yet many older adults continue to experience unmet needs. While individual-level factors are better-studied, less is known about how neighborhood-level place-based factors (PBFs, e.g., poverty, housing conditions, transportation, and internet access) shape access to and adequacy of HCBS. This study addresses that gap by examining the added explanatory value of PBFs in predicting HCBS use and unmet needs. We analyzed data from 6558 community-dwelling adults aged ≥ 65 years using the 2022–2023 National Core Indicators–Aging & Disability Adult Consumer Survey. Outcomes included use of six HCBS types, consumer-reported unmet needs for each type, and overall unmet HCBS needs. PBFs were measured at the ZIP code level using the 2016–2020 American Community Survey. Nested logistic regression models estimated incremental variance (McFadden’s R^2^) explained by PBFs, adjusting for individual demographics, health status, state, and proxy response. Adding PBFs increased explained variance by 7.98–22.70% for HCBS use, 35.92–48.00% for unmet needs by service type, and 51.85% for overall unmet HCBS needs. PBFs meaningfully influence both access to and adequacy of HCBS. Using PBFs to guide resource allocation and targeting modifiable PBFs could improve HCBS access and efficiency.

## 1. Introduction

Home- and community-based services (HCBS) are a cornerstone of the long-term services and supports (LTSS) system in the United States (U.S.) [1]. HCBS encompass a range of services such as personal care, transportation, home-delivered meals, homemaker services, and adult day programs, which support older adults and individuals with disabilities to live safely and independently in their homes and communities [1,2]. HCBS are funded through a variety of public sources, including Medicaid waivers and state-funded programs, and they have been shown to promote autonomy and reduce reliance on institutional care [1]. As the U.S. population ages and demand for community-based alternatives to nursing home care increases, ensuring equitable access to high-quality HCBS has become a national policy priority [1,2].

Despite growing investment in HCBS, longstanding disparities in service access and quality persist [2,3,4]. These disparities are shaped not only by individual-level factors such as age, disability status, income, and race/ethnicity but also by broader social and structural determinants of health [2,3,4]. Place-based factors (PBFs), i.e., characteristics of the physical, economic, and social environments in which individuals live, have emerged as critical, yet under-examined determinants of HCBS access and utilization [5,6]. For example, neighborhood-level poverty, housing quality, access to transportation, and broadband connectivity can facilitate or constrain an individual’s ability to access HCBS, engage with service providers, and maintain community integration [5,6,7].

Prior research on social determinants of health (SDOH) and health-related social needs (HRSNs) has similarly demonstrated that including location-based identifiers meaningfully improves the amount of variance explained by the model [6,7]. Including location identifiers has also revealed differences that are otherwise obscured, highlighting the value of neighborhood context in shaping health and service outcomes [6,7]. These findings provide a precedent for examining how PBFs may enhance our understanding of HCBS access and adequacy. While previous studies have documented geographic variation in HCBS availability and spending across states and counties [1], few have examined how neighborhood-level PBFs relate to unmet service needs from the perspective of consumers [5,6,7]. In one study conducted in the state of Pennsylvania, the addition of place-based factors significantly improved the model fit and explained 54% of variance in consumers’ self-reported unmet needs [7]. However, these findings may not be generalizable outside the state where the study was conducted. Consumers’ self-reported unmet service needs are a key quality indicator of performance and equity in publicly funded HCBS [1,2,3,4,5,8]. They are associated with an increased risk of hospitalization, caregiver burden, institutionalization, and lower quality of life [8]. Understanding how PBFs influence consumer-reported unmet HCBS needs is critically important for HCBS quality assurance.

Our study investigates the role of ZIP code-level PBFs in HCBS use and consumer-reported unmet service needs among older adults receiving publicly funded HCBS. Using data from the 2022–2023 National Core Indicators–Aging & Disability (NCI-AD) Adult Consumer Survey (ACS) linked to ZIP code-level PBFs from the 2019 American Community Survey, we assess how much additional variance in HCBS use and unmet HCBS needs is explained by the inclusion of PBFs in regression models that already account for individual demographic and health-related factors. Our findings aim to deepen the understanding of the structural factors contributing to variation in HCBS access and to inform policy and programmatic strategies that target modifiable community-level barriers to accessing HCBS [1,2].

### 1.1. Conceptual Framework

Guided by a conceptual framework which integrates the Socio-Ecological Model with the Social Determinants of Health (SDoH) paradigm, our analyses situate home- and community-based service (HCBS) use and unmet need within a multilayered context that extends beyond individual attributes [9,10].

The Socio-Ecological Model emphasizes that health behaviors and outcomes arise from nested spheres of influence—individual, interpersonal, community, and policy environments [9]. Complementing this, the SDoH framework highlights how structural factors such as poverty, housing instability, and digital exclusion create uneven distributions of risk and resources [10]. Alzheimer’s disease and related dementias (ADRD) exemplify how these domains intersect: at the individual level, cognitive decline heightens reliance on formal HCBS and informal caregivers; at the interpersonal level, caregiver burden may be exacerbated by inadequate respite services; and at the community level, neighborhood walkability, transportation options, and availability of dementia-capable providers may determine the feasibility of aging in place [11]. State-level policies, ranging from Medicaid waiver dementia add-ons to dementia-friendly community initiatives, might further modulate service access and quality [1]. By integrating these models, we conceptualize PBFs *as potentially modifiable or intervenable community- and policy-level levers* that shape HCBS engagement. ZIP code indicators from the 2016–2020 American Community Survey serve as proxy indicators for several SDOH domains such as economic (e.g., poverty), education, neighborhood and environment (e.g., crowded housing, transportation, ZIP code-based rural–urban classification), access to healthcare services (e.g., broadband access for telehealth; availability of social services for older adults and adults living with disabilities), and social context (e.g., demographic composition of community), in which older adults with and without ADRD must navigate service systems [5,6]. We hypothesize that such contextual attributes explain variance in both HCBS use and unmet needs beyond individual-level characteristics. This dual-model framework informs our nested regression strategy: quantifying the incremental explanatory power of PBFs illuminates how community environments, shaped by broader social and policy forces, either facilitate or hinder equitable realization of person-centered HCBS goals, particularly for those living with ADRD. We did not select other frameworks such as the OASIS (Outcome and Assessment Information Set) framework. While well-validated for home healthcare quality monitoring, the OASIS framework is narrower in scope. It is primarily designed for clinical assessment of individual patients receiving Medicare home health services, emphasizing functional status, clinical conditions, and care outcomes. OASIS does not capture broader structural and contextual determinants of service access (particularly community-level factors such as neighborhood poverty or infrastructure) which were central to our research question. Therefore, the SDOH paradigm was a better fit for our study objectives, allowing us to situate consumer-reported HCBS outcomes within a framework that explicitly incorporates both individual and structural (i.e., place-based) drivers of unmet needs [10].

## 2. Methods

Our research received approval from the Institutional Review Board at the University of Minnesota.

### 2.1. Data Source and Survey Methods

We analyzed data from the NCI-AD ACS, an annual cross-sectional survey, which provides a comprehensive assessment of HCBS consumers’ self-reported care experiences, satisfaction with services, and quality of life; health-related outcomes; and the effectiveness of HCBS in promoting independence and autonomy [4,8]. Participation is voluntary, and states can choose which waiver programs to include in the sampling. The NCI-AD ACS includes two main components: a Consumer Survey section and a Background Information (BI) section. The Consumer Survey captures the perspectives of individuals receiving LTSS and assesses multiple domains, including overall care experience, satisfaction with services, quality of life, health-related outcomes, and the perceived effectiveness of LTSS in fostering independence and autonomy. In contrast, the BI section compiles participant data primarily from administrative sources, as detailed below. Until 2019, prior to the onset of the COVID-19 pandemic, the survey was administered exclusively in person. Starting with the 2021–2022 administration cycle, however, the survey protocol was adapted to include remote administration methods such as telephone interviews, following a successful pilot validation study. Survey administrators may permit the use of proxy respondents for certain items when the primary respondent is unable to complete the consumer portion independently.

Participating states are responsible for defining the sampling frame, typically drawing on specific LTSS funding programs. They apply probabilistic sampling methods to select participants from the eligible population, targeting a maximum margin of error of 5% at a 95% confidence interval. Prior to initiating interviews, a detailed set of background data is assembled for each respondent in the BI section. Information captured in the BI section (comprising information such as race, sex, diagnosis of Alzheimer’s disease or related dementias, mental health conditions, and other disabilities) is predominantly derived from state-level administrative databases. These may include case management systems, managed care records, and Medicaid billing data. Service-related information, such as the type of HCBS utilized, is generally obtained from administrative systems like the Medicaid Management Information System (MMIS) rather than relying on consumer self-report. Sample selection typically begins three to four months following the start of the annual survey wave. However, due to the protracted nature of data collection, interviews may be conducted as late as eight months after sample identification. The BI section continues to rely heavily on state administrative data to provide demographic and service utilization information. In instances where administrative records are incomplete or unavailable, participants may be asked to supply the necessary details at the end of the survey. Specific elements of the BI section, such as the primary LTSS funding source, the main service program, types of services received, length of service participation, status of self-direction, and whether the individual has a legal guardian, all must be verified using administrative data sources. To be eligible for inclusion in the NCI-AD ACS, individuals must be actively receiving at least one LTSS (e.g., personal care, homemaker services, or transportation) at a minimum frequency of twice per week for a duration of approximately three months. This level of active service utilization is a prerequisite for the NCI-AD ACS participation.

### 2.2. Study Sample

**Figure 1:**
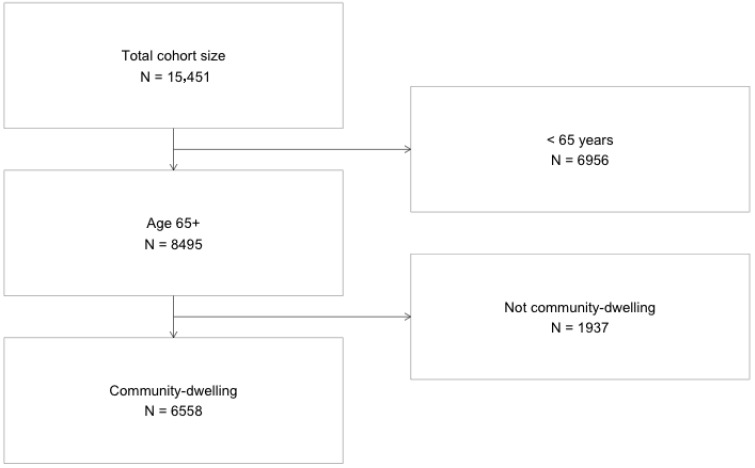
Participant selection flow diagram: The National Core Indicators—Aging & Disability Survey 2022–2023.

Our study includes community-dwelling NCI-AD ACS respondents aged 65 years or more who use publicly funded HCBS. Therefore, from a total sample of 15,451 respondents from 18 states, who participated in the NCI-AD ACS 2022–2023 wave, we excluded 6956 respondents under the age of 65 years and further excluded 1937 respondents who resided in institutional care settings (e.g., nursing home residents), yielding a final analytic sample of 6558 community-dwelling older adult consumers of HCBS [Figure 1].

### 2.3. Ascertainment of Main Outcomes: HCBS Use and Consumer-Reported Unmet HCBS Needs

Our analysis concentrated on six of the most frequently utilized HCBS: (1) personal care assistance, (2) homemaker or chore support, (3) home-delivered meals, (4) adult day programs, (5) transportation services, and (6) caregiver respite or support services. Information regarding participants’ use of these services was obtained from the BI section of the NCI-AD ACS. Within this section, service use was identified based on the response to the question, “What type of paid long-term care support is the individual receiving?” Respondents could select multiple services from a predefined list of HCBS categories (presented in Supplemental Table S1). Based on this information, individuals were classified as users or non-users of each specific service, which served as our study’s primary outcomes.

To determine whether participants had unmet needs related to their HCBS, we reviewed responses to a survey item from the consumer-reported portion of the instrument that asked, “Do the long-term care services you receive meet your current needs and goals?” Respondents who answered “no” or “some” were categorized in our analysis as having overall unmet service needs, which served as our study’s primary outcome.

Individuals indicating unmet needs were then presented with a follow-up item: “What additional long-term care services might help you meet your needs and goals?” In this follow-up, participants could indicate one or more services from the same set of six core HCBS that they believed would help meet their needs. Their responses to this item were used to identify unmet needs for specific types of HCBS. Using this item, we ascertained consumer-reported unmet service needs for the aforementioned six HCBS, as secondary outcomes.

### 2.4. Ascertainment of Place-Based Factors (Independent Variables)

The American Community Survey is an ongoing, nationally representative survey administered by the U.S. Census Bureau to provide detailed, annually updated information on the demographic, social, economic, and housing characteristics of the U.S. population [12]. Unlike the decennial census, the American Community Survey is conducted continuously, collecting data monthly from approximately 3.5 million households each year. This enables timely, granular estimates that inform federal, state, and local policy decisions, resource allocation, and program planning. The 5-year estimates from the 2016–2020 American Community Survey, used in this study, aggregate responses across five calendar years to enhance statistical reliability, especially for smaller geographic units such as ZIP Code Tabulation Areas. The American Community Survey employs a stratified, address-based sampling design and collects responses via mail, telephone, internet, and in-person interviews to ensure broad coverage and reduce nonresponse bias. For this analysis, we extracted ZIP code-level indicators representing PBFs relevant to older adults’ access to and need for HCBS, including measures of poverty, educational attainment, housing cost burden, transportation access, and internet connectivity. These indicators serve as proxies for structural and social determinants of health within communities and provide critical context for understanding geographic variation in HCBS utilization and unmet needs among older adults.

We ascertained ZIP code-level summary estimates of several PBFs selected based on our conceptual model described above (in Section 1.1) as well as data availability. These included the following:

1. *Education*: (i) Percent population who are high school dropouts (includes people of compulsory school attendance age or above who were not enrolled in school and were not high school graduates).
2. *Economic stability*: (i) Percent non-employed for population aged 16–64 years [measured as (not in labor force + unemployed between 16 and 64 years)/(civilian + not in labor force between 16 and 64 years)]. (ii) Percent population in households earning less than 100% of the federal poverty line. We included poverty among adults aged < 65 years as a ZIP code-level indicator of economic stability because this measure, while not specific to older adults, reflects broader community economic conditions that influence the availability of informal caregivers and local workforce capacity; factors that indirectly affect HCBS access and adequacy for the older population.
3. *Social context:* (i) Percent population white, Black, and Hispanic in the area. (ii) Percent population foreign-born (i.e., born outside the United States). (iii) Percent single-parent families with dependents aged < 18 years. (iv) Percent “high-needs” population (includes proportion of residents in an area who are aged 65 years and older, women, or children < 5 years).
4. *Neighborhood and physical environment*: (i) Percent households living in crowded housing units [measured as (tenure by occupants per room − (owner-occupied + renter-occupied))/(total occupied housing units)]. (ii) Percent households with no vehicle (as an indicator of access to transportation).
5. *Healthcare service access*: As an indicator for telehealth capability, we evaluated 2 PBFs—internet access and broadband service coverage: (i) Internet access was determined by asking if any member of the household has access to the internet; “access” refers to whether or not someone in the household uses or can connect to the internet, regardless of whether or not they pay for the service. (ii) Similarly access to broadband services was asked in a follow-up question to internet access, with broadband (high-speed) internet service referring to cable, fiber optic, or DSL (digital subscriber line) mode of high-speed internet service delivery. (iii) In regard to the availability of social services, we ascertained the median total number of all social services available in an area and median total number of elderly/disabled social services available in the entire ZIP code, available per square mile within the ZIP code, and per square mile within the ZIP code including only those services with at least one consumer.

Additionally, we evaluated the consumer’s location as a PBF, using the Rural-Urban Continuum Area Classification (RUCA) based on ZIP codes. From the BI section in the NCI-AD ACS, we ascertained a geographic classification of the individual’s ZIP code (metropolitan, micropolitan, small town, or rural). Of note, we used ZIP code data because in our datasets, ZIP code is the smallest available geographic unit for PBFs, so we expect a similar distribution of PBFs within the ZIP code and assume that there is no variation within zip codes.

### 2.5. Covariates

We selected person-level demographic and health-related factors as covariates based on prior research studies [3]. We ascertained demographic characteristics such as age; gender (categorized as male or female); racial/ethnic identification (non-Hispanic Black, non-Hispanic White, or other racial/ethnic groups); having a legal guardian; and living arrangement (residing alone, with family members, or with non-family individuals). Additionally, we accounted for the state of residence. We also included variables indicating whether the participant was enrolled in Medicare and whether survey responses were provided directly by the individual or via a proxy. Health and functional status were captured through multiple indicators: self-reported need for assistance with activities of daily living (categorized as requiring “a lot” or “some” help versus “no help”); self-rated health, originally collected on a five-point Likert scale but grouped into three categories for analysis—poor/fair, good, and very good/excellent; and presence or absence of specific clinical conditions including dementia, physical disability, brain injury, and mental illness, which were documented in the BI section of the survey and were coded as binary variables (yes/no) in our models. We also included a binary indicator for whether each respondent has more than one of the following chronic conditions: dementia, physical disability, brain injury, and mental illness.

### 2.6. Statistical Analysis Plan

We summarized descriptive characteristics for PBFs as percentage proportions at the ZIP code level, descriptive sociodemographic and health-related characteristics of NCI-AD ACS participants, and the outcome variables including HCBS use and unmet HCBS needs for six HCBS as well as overall unmet HCBS need. All descriptive summary estimates were stratified at two levels: (1) first, we stratified by dementia status (with versus without a dementia diagnosis); and (2) within the two groups by dementia status, we further stratified summary estimates by RUCA codes (metropolitan, micropolitan, small towns, and rural areas). Micropolitan areas, as defined by RUCA, are communities with an urban cluster of 10,000 to 49,999 residents and adjacent territory with strong commuting ties to that cluster. They represent areas that are more urbanized than rural communities but smaller and less densely populated than metropolitan areas. We stratified the analyses by dementia status given the distinct care needs, service utilization patterns, and heightened vulnerability to unmet needs among older adults living with dementia, as indicated in the explanation of the conceptual framework [11].

For each outcome variable, we estimated three serial nested logistic regression models. Model 1 adjusted for dementia status, state (location of the participant), and proxy response (vs. self-response); model 2 further adjusted for person-level attributes including sociodemographic and health-related variables; and model 3 further adjusted for PBFs. To compare the nested models (i.e., model 3 vs. 2 and model 2 vs. 1), we calculated the Akaike Information Criterion (AIC) (for model fit) and McFadden’s R^2^ (for calculating incremental variance in outcomes explained in each successive expanded regression model) [13,14]. To address missing data in the variables mentioned above, we employed multiple imputation by chained equations (MICE). We pooled results, including the AIC and McFadden’s R^2^, from the 20 imputed datasets. All analyses were performed using R version 4.4.0 (R Core Team, 2024).

## 3. Results

Place-based characteristics (ascertained by the 2016–2022 American Communities Survey) varied notably by both rurality and dementia status among older adult consumers of HCBS (Table 1). ZIP codes (ascertained from the NCI-AD ACS) in rural and small-town areas had consistently higher proportions of residents with high social needs and lower racial and ethnic diversity, particularly with a predominance of white residents (up to 92% in rural areas) and very small proportions of Black and Hispanic individuals. These patterns were consistent across dementia status. PBFs indicating social vulnerability, such as poverty, dropout rates, and unemployment, were relatively stable across geographies, though slightly elevated in micropolitan and small-town areas. Availability of services showed stark urban–rural gradients: the median number of all social services was highest in metropolitan ZIP codes (34 for those without dementia; 32 for those with dementia) and dropped sharply in rural areas (2 services), with a near absence of services per square mile in small-town and rural areas. Similarly, services specifically for elderly or disabled persons were rare outside metropolitan areas. Broadband and internet access followed similar gradients, with the lowest access in rural areas regardless of dementia status.

Participant characteristics (ascertained by the NCI-AD ACS 2022–2023 wave) revealed distinct patterns by both rurality and dementia status (Table 2). Individuals with dementia were consistently older than their counterparts without dementia, with median ages ranging from 79 to 81 years across geographies, compared to a consistent median of 74 years among those without dementia. Women comprised the majority across all groups, with the highest proportion observed among individuals with dementia living in small towns (80%). Multiple chronic conditions were markedly more prevalent among individuals with dementia, affecting over two-thirds of this group across all geographies (67–81%) compared to only 19–29% of those without dementia. This is unsurprising given that dementia itself counts as a chronic condition, meaning that patients with dementia only needed one additional chronic condition to count as having multiple chronic conditions. Living arrangements differed notably: while over 60% of those without dementia lived alone, most individuals with dementia lived with family members, particularly in metropolitan and rural areas (61% and 63%, respectively). The racial and ethnic composition showed substantial geographic variation, with metropolitan areas housing more racially and ethnically diverse participants (including a higher proportion of Black and Hispanic individuals) than rural areas, where white individuals predominated (>80%). Self-reported health was generally rated as poor or fair across groups, with a slight trend towards better ratings among rural participants. Proxy respondents were far more common among individuals with dementia (ranging from 46% to 66%), underscoring potential cognitive and functional challenges.

Across all geographic areas, older adults with and without dementia reported varying levels of service use and unmet needs for HCBS, as measured by the NCI-AD ACS 2022–2023 wave (Table 3). Personal care services were the most frequently used service overall, particularly among individuals with dementia in metropolitan areas (43%), followed by those without dementia in metropolitan areas (40%). Homemaker services showed an inverse trend: use was more common in rural areas, especially among individuals without dementia (36%) compared to those with dementia (26%). Use of adult day services was consistently low, but notably higher among individuals with dementia, especially in metropolitan areas (10%) compared to other groups. Consumers’ self-reported unmet service needs for personal care were generally higher among individuals with dementia, reaching 14% in micropolitan areas, compared to 10% or lower in individuals without a dementia diagnosis across the rural–urban continuum. Transportation services were used infrequently, though unmet transportation needs were consistently higher among those without diagnosed dementia, in metropolitan areas and small towns.

**Figure 2:**
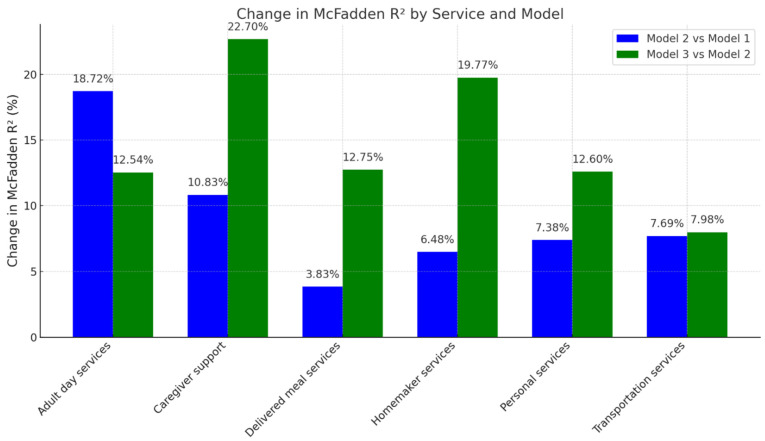
Incremental variance in service use explained by place-based factors among older adult consumers of publicly funded home- and community-based services: The National Core Indicators—Aging & Disability Survey 2022–2023. Note: Model 1 is adjusted for dementia status, state (location of the participant), and proxy response (vs. self-response). Model 2 is adjusted for Model 1 covariates + person-level attributes including sociodemographic and health-related variables. Model 3 is adjusted for Model 2 covariates + PBFs.

For the outcome of HCBS use, model fit improved progressively across all services with the inclusion of person-level and place-based factors, as evidenced by reductions in AIC and increases in McFadden’s R^2^ (Table 4; Figure 2). For each service, Model 3, which adjusted for dementia, sociodemographic and health characteristics, and place-based factors, demonstrated the best fit and highest explanatory power. The largest incremental gain in explained variance attributable to place-based factors was observed for caregiver support services, with a 22.7% increase in McFadden’s R^2^ between Models 2 and 3. Homemaker services also showed substantial gains (19.8%), followed by delivered meals (12.8%), adult day services (12.5%), personal services (12.6%), and transportation (8.0%). Coefficient estimates from the regression models are shown in Supplemental Tables S2–S4.

**Figure 3:**
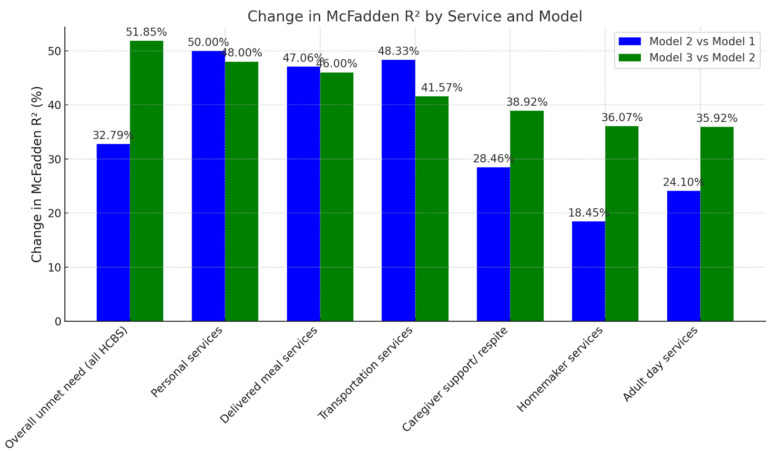
Incremental variance in consumer-reported unmet service needs explained by place-based factors among older adult consumers of publicly funded home- and community-based services: The National Core Indicators—Aging & Disability Survey 2022–2023. Note: Model 1 is adjusted for dementia status, state (location of the participant), and proxy response (vs. self-response). Model 2 is adjusted for Model 1 covariates + person-level attributes including sociodemographic and health-related variables. Model 3 is adjusted for Model 2 covariates + PBFs.

For the outcome of consumer-reported unmet HCBS needs, across all six types of HCBS, model fit improved with the sequential inclusion of person-level and place-based factors, as reflected in decreasing AIC values and an increasing McFadden’s R^2^ (Table 5; Figure 3). The largest gains in explained variance for consumer-reported unmet needs were observed in the full models (Model 3), which included place-based factors. For overall unmet HCBS needs, Model 3 explained 51.9% more variance than Model 2, highlighting the significant influence of geographic context. Similar substantial gains were seen for personal services (48.0%), delivered meals (46.0%), and transportation (41.6%). Although person-level factors accounted for notable increases in the explained variance across services (e.g., 50.0% for personal services from Model 1 to Model 2), the further inclusion of place-based variables consistently improved model performance and led to an increase in the variance in unmet needs explained by PBFs. Estimates from the regression models are shown in Supplemental Tables S5–S7.

## 4. Discussion

Our study examines how PBFs affect HCBS use and consumer-reported unmet service needs among older adults receiving publicly funded HCBS in the United States. While most prior work has focused on individual factors [5,6], we quantified the additional variance in HCBS use and unmet needs explained by PBFs after adjusting for individual demographics and health status. By highlighting these structural drivers, our findings offer insights for international policymakers and practitioners aiming to identify and modify community-level barriers to equitable HCBS delivery and to design interventions that strengthen aging-in-place supports [6].

Findings from our analyses of the 2016–2020 American Communities Survey data revealed persistent place-based disparities in service infrastructure and sociodemographic composition. Rural areas—where service needs may be greatest—consistently face limited availability of supportive resources. Our findings align with previously reported epidemiologic trends in various SDOH, including economic stability, education, neighborhood and physical environment, healthcare service access, and social context, as outlined in our conceptual framework. Rural areas continue to face substantial barriers due to limited service infrastructure, despite potentially higher levels of need. Prior studies have shown that older adults in rural areas are more likely to experience functional limitations and chronic conditions, yet have less access to HCBS, including transportation, personal care, and adult day services [15]. Our analysis supports these findings, demonstrating that rural communities have fewer social services per square mile and fewer specialized services for older adults and individuals with disabilities. These findings suggest that the sociodemographic vulnerabilities common in rural areas, such as higher proportions of older adults and lower socioeconomic status, may be compounded by limited local capacity to meet complex care needs [15].

In our analysis of the NCI-AD ACS 2022–2023 wave, we found demographic and health-related differences between older adults with and without dementia, as well as the persistent influence of rurality on living conditions, support networks, and health profiles. Our analyses also indicate both rural and dementia status-related disparities in HCBS utilization and unmet HCBS needs, with individuals with dementia often reporting higher levels of unmet needs despite slightly higher rates of service use for some services. Our findings echo prior research highlighting the complex interplay between dementia, rurality, and access to HCBS [16,17]. Older adults with dementia were older, had more chronic conditions, and were less likely to live alone, which is consistent with prior studies showing a greater reliance on informal caregiving in this population [11,15]. Rural residence further compounded social vulnerabilities, with individuals in these settings experiencing more limited support networks and poorer health [18]. Notably, despite slightly higher utilization of some HCBS among people with dementia, unmet needs remained disproportionately high in our study sample (particularly for personal care and homemaker services), underscoring the inadequacy of the current service capacity to meet the complex care demands associated with dementia [11,15]. These findings are consistent with the literature documenting rural gaps in dementia-capable services [15,16] and highlight the urgency of developing more responsive, dementia-informed HCBS systems in under-resourced areas [11].

Our regression analyses reveal that place-based factors (PBFs) substantially enhance the explanation of variation in HCBS use and consumer-reported unmet needs, beyond what can be accounted for by individual-level characteristics alone. This added explanatory power is particularly notable for services such as caregiver support and homemaker assistance, which are highly dependent on local service infrastructure, workforce availability, and administrative capacity, as reported in the prior literature [19,20,21,22,23]. These findings align with broader health services research demonstrating the predictive value of neighborhood-level contextual factors. For instance, Kind et al. (2014) linked the Area Deprivation Index (ADI) at the census block group level to an increased 30-day rehospitalization risk among Medicare beneficiaries—finding that residents of the most disadvantaged neighborhoods had a 9% higher risk, comparable in magnitude to chronic disease predictors [24]. Similarly, Lusk et al. (2023) found that neighborhood socioeconomic deprivation (measured via ADI) independently predicted 30-day mortality among critically ill patients—even after adjusting for individual poverty, comorbidities, access to care, and facility characteristics [25]. These studies reinforce that area-based deprivation measures add meaningful explanatory power in health outcomes beyond individual-level factors. Prior research has documented rural–urban disparities in HCBS access, with rural areas often facing long waitlists, fewer provider options, and transportation barriers [1,15,26,27]. For example, older adults in far rural or remote regions may rely heavily on family caregivers due to a lack of formal service providers, leading to a greater caregiver burden and unmet needs [15,16]. Our findings align with studies emphasizing the influence of county-level health service density, Medicaid waiver generosity, and local agency capacity on service use and equity [6,28,29,30]. The observed gains in model fit and variance explained by adding PBFs underscore their importance as structural determinants of service access, which are not always visible in person-level datasets. Collectively, these comparisons illustrate that PBFs consistently show added value in modeling health and service outcomes across diverse contexts. Our results both corroborate these prior findings and extend them by showing for the first time that neighborhood-level factors strongly influence the consumer-reported adequacy of HCBS. These results highlight the importance of geographically responsive policy solutions that recognize the uneven geography of HCBS provision, particularly for vulnerable populations such as people living with dementia and their caregivers, who may experience compounding risks in under-resourced areas [6,30].

While aging in place remains a central goal of long-term care policy, scholars have highlighted that place-based factors (PBFs) can both enable and constrain the achievement of this objective. Golant (2015) and Buffel (2023) note that neighborhood disadvantage, limited transportation, and poor housing quality may hinder older adults’ ability to age safely and independently [31,32]. This aligns with critiques emphasizing that aging in place may not always be feasible or desirable if the environment is unsupportive, suggesting the importance of “aging in the right place” [33,34,35]. Our findings contribute to this emerging perspective by demonstrating that ZIP code-level PBFs meaningfully influence HCBS use and unmet needs, highlighting the potential for targeted resource allocation to facilitate optimal aging environments. Additionally, our findings complement and extend existing place-based interventions aimed at supporting older adults, such as the WHO Age-Friendly Cities framework and the CDC’s Healthy Aging Initiative, by providing empirical evidence on how ZIP code-level, place-based factors influence HCBS use and unmet needs [34,35,36,37]. These initiatives emphasize modifying physical, social, and service environments to enhance aging in place, which aligns with our results showing that neighborhood characteristics meaningfully shape service access and adequacy. By identifying specific PBFs associated with unmet needs, our study can inform the targeting and prioritization of these interventions, ensuring resources are directed to areas with the greatest gaps. Ultimately, integrating our findings with these programs can enhance the effectiveness of community-level strategies and support more equitable access to home- and community-based services. Our recommendations align with 2025 U.S. policy priorities emphasizing equity in home- and community-based services, including initiatives to expand Medicaid HCBS access and integrate social determinants into care planning [10]. Future research could extend these analyses internationally by examining place-based factors in aging populations across countries with diverse long-term care systems, such as the Netherlands, Japan, and the U.K., to identify cross-national strategies for optimizing service access and reducing unmet needs [32,36].

### Limitations

While our work addresses an important gap, we acknowledge several limitations in our study. First, there are limitations of the study design. The observational and cross-sectional design precludes causal inference and limits our ability to establish temporality between dependent variables and predictor variables. Although our dataset provides information on service utilization, it does not include granular details about individuals’ care plans or the types of caregivers involved in service delivery. Furthermore, there could be residual and unmeasured confounding from variables such as frailty. We chose an age threshold of 65 years based on administrative norms for the CMS (benefits for older adults versus younger adults with disabilities); however, this cut-off is artificially imposed and can impact generalizability to the entire older adult population in society.

Second, the NCI-AD ACS also has several methodological limitations. Participation is voluntary for states, and although the program mandates standardized sampling protocols—including minimum sample sizes and margins of error ≤ 5%—states may adapt their sampling to align with local priorities. Consequently, findings may not be generalizable to older adults in non-participating states or to the broader national population of long-term services and supports (LTSS) users. Additionally, background data availability varies across states. We addressed missing data using multiple imputation (MICE) and found consistent results with complete-case analyses.

Third, we analyzed the NCI-AD ACS data collected during the COVID-19 pandemic-related public health emergency period. Although all quarantine-related restrictions had been lifted, social distancing was still widely advised and prominently followed, which may have impacted HCBS use and unmet needs. Future research should confirm whether our findings persist after the end of the public health emergency period in May 2023.

Fourth, our datasets and data use agreement for the NCI-AD allow analysis only at the ZIP code level as the smallest geographic unit, thus assuming homogeneity of place-based factors within the unit. Future studies should generate data for smaller geographic units at the neighborhood level to allow more granular analyses for an improved validity of findings.

Finally, the goal of our study was to evaluate the overall contribution of PBFs in explaining the variance in HCBS use and unmet HCBS needs. We did not interpret associations of individual PBFs in depth as this was beyond the scope of our study, and we anticipated a lack of power due to the reliance on a single survey wave, as confirmed by the wide confidence intervals of estimates shown in the Supplemental Material. Future studies should investigate individual PBFs that showed promising associations in our analyses, by devising larger, well-designed, bespoke studies for individual PBFs.

## 5. Conclusions

Overall, the findings from our study of the NCI-AD ACS data from community-dwelling older adult consumers of publicly funded HCBS in the U.S. indicate both dementia status-related and geographic disparities across the rural–urban continuum in HCBS utilization and consumers’ self-reported unmet HCBS needs, with individuals with dementia often reporting higher levels of unmet needs despite slightly higher rates of service use for some services. Our analyses highlight that PBFs have a considerably large influence on the variance in HCBS use and consumer-reported unmet HCBS needs. Integrating PBFs into predictive analytics models can enhance the accuracy and equity of health outcome forecasts by capturing the broader social and environmental contexts influencing individual health. While traditional models, such as logistic regression, primarily utilize clinical data, incorporating PBFs allows for a more comprehensive understanding of health disparities and can inform targeted interventions. However, challenges such as data standardization, ethical considerations, and the need for interdisciplinary collaboration must be addressed to fully realize the potential of these models in improving health outcomes and reducing disparities [38]. Future research in HCBS quality assurance should incorporate PBFs into their analyses and design robust causal studies to help identify modifiable interventions targeting PBFs, to improve HCBS access and quality.

## Figures and Tables

**Table 1:** Summary estimates of ZIP code-level place-based factors stratified by rurality and dementia status: The American Communities Survey 2016–2020. Values are median (IQR) across individuals by dementia status and ZIP code classification.

Place-Based Factors	No Dementia				Dementia			
	Metropolitan N = 3251	Micropolitan N = 685	Small Town N = 404	Rural N = 270	Metropolitan N = 643	Micropolitan N = 117	Small Town N = 55	Rural N = 31
% Consumers with high social needs (includes older adults of age 65 years or more, women, and children) *	0.21 (0.19, 0.24)	0.24 (0.21, 0.26)	0.26 (0.23, 0.28)	0.28 (0.24, 0.32)	0.21 (0.19, 0.23)	0.24 (0.21, 0.26)	0.26 (0.24, 0.28)	0.28 (0.24, 0.31)
% Black *	0.09 (0.03, 0.30)	0.02 (0.01, 0.05)	0.01 (0.00, 0.03)	0.00 (0.00, 0.01)	0.09 (0.03, 0.26)	0.02 (0.00, 0.04)	0.01 (0.00, 0.05)	0.00 (0.00, 0.01)
% Hispanic	0.07 (0.04, 0.14)	0.05 (0.02, 0.12)	0.04 (0.02, 0.18)	0.03 (0.01, 0.07)	0.08 (0.03, 0.14)	0.06 (0.02, 0.14)	0.03 (0.02, 0.08)	0.04 (0.02, 0.06)
% White *	0.64 (0.40, 0.81)	0.85 (0.75, 0.92)	0.86 (0.68, 0.93)	0.92 (0.81, 0.96)	0.65 (0.39, 0.80)	0.83 (0.74, 0.91)	0.89 (0.72, 0.94)	0.92 (0.79, 0.94)
% Foreign-born *	0.07 (0.03, 0.14)	0.03 (0.01, 0.06)	0.02 (0.01, 0.04)	0.01 (0.00, 0.02)	0.08 (0.04, 0.15)	0.03 (0.01, 0.06)	0.02 (0.01, 0.04)	0.01 (0.00, 0.02)
% Single-parent household	0.16 (0.10, 0.22)	0.14 (0.11, 0.17)	0.13 (0.10, 0.18)	0.09 (0.06, 0.14)	0.14 (0.10, 0.21)	0.16 (0.13, 0.19)	0.12 (0.09, 0.16)	0.11 (0.06, 0.15)
% Drop-out	0.10 (0.06, 0.15)	0.12 (0.08, 0.15)	0.11 (0.08, 0.15)	0.11 (0.07, 0.15)	0.10 (0.06, 0.15)	0.11 (0.09, 0.19)	0.11 (0.08, 0.14)	0.09 (0.07, 0.14)
% Below poverty	0.13 (0.09, 0.21)	0.15 (0.11, 0.19)	0.15 (0.11, 0.19)	0.13 (0.09, 0.19)	0.11 (0.07, 0.18)	0.15 (0.11, 0.21)	0.14 (0.11, 0.19)	0.11 (0.07, 0.19)
% Non-unemployed	0.35 (0.32, 0.40)	0.41 (0.36, 0.45)	0.42 (0.38, 0.48)	0.44 (0.38, 0.52)	0.34 (0.31, 0.38)	0.39 (0.36, 0.45)	0.43 (0.37, 0.49)	0.46 (0.36, 0.50)
% Unemployed	0.053 (0.038, 0.072)	0.049 (0.036, 0.061)	0.043 (0.029, 0.060)	0.043 (0.021, 0.065)	0.050 (0.036, 0.068)	0.048 (0.036, 0.067)	0.047 (0.034, 0.064)	0.033 (0.020, 0.073)
% Crowded housing	0.021 (0.012, 0.034)	0.017 (0.010, 0.027)	0.017 (0.009, 0.032)	0.014 (0.004, 0.027)	0.020 (0.012, 0.035)	0.020 (0.012, 0.034)	0.020 (0.010, 0.031)	0.012 (0.002, 0.033)
% Household with no car	0.07 (0.04, 0.12)	0.06 (0.05, 0.08)	0.06 (0.04, 0.09)	0.05 (0.03, 0.07)	0.06 (0.04, 0.11)	0.06 (0.05, 0.08)	0.05 (0.03, 0.08)	0.05 (0.03, 0.10)
Number of all social services available *	34 (18, 56)	28 (10, 43)	11 (6, 17)	2 (1, 5)	32 (17, 53)	23 (12, 38)	13 (6, 21)	2 (1, 4)
Number of social services per square mile, which have been purchased at least once *	2.5 (0.6, 5.6)	0.2 (0.1, 0.3)	0.0 (0.0, 0.1)	0.0 (0.0, 0.0)	2.3 (0.6, 4.7)	0.2 (0.1, 0.2)	0.1 (0.0, 0.1)	0.0 (0.0, 0.0)
Number of elderly/disabled person services	2 (1, 4)	2 (1, 4)	1 (0, 2)	0 (0, 1)	2 (1, 4)	2 (0, 4)	1 (0, 1)	1 (0, 1)
Number of elderly/disabled person services per square mile, which have been purchased at least once *	0.09 (0.01, 0.37)	0.01 (0.00, 0.03)	0.00 (0.00, 0.01)	0.00 (0.00, 0.00)	0.09 (0.01, 0.33)	0.01 (0.00, 0.03)	0.00 (0.00, 0.01)	0.00 (0.00, 0.01)
Internet access	0.86 (0.80, 0.90)	0.81 (0.78, 0.84)	0.79 (0.74, 0.82)	0.77 (0.71, 0.81)	0.87 (0.81, 0.92)	0.81 (0.78, 0.84)	0.79 (0.75, 0.82)	0.79 (0.69, 0.82)
Broadband access	0.85 (0.79, 0.90)	0.81 (0.78, 0.84)	0.79 (0.74, 0.82)	0.77 (0.71, 0.80)	0.87 (0.80, 0.91)	0.81 (0.78, 0.84)	0.78 (0.74, 0.81)	0.78 (0.69, 0.81)

* *p* < 0.05.

**Table 2:** Descriptive characteristics of participants, stratified by rurality and dementia status: The National Core Indicators—Aging & Disability Survey 2022–2023.

Characteristics	No Dementia				Dementia			
	Metropolitan N = 3251	Micropolitan N = 685	Small Town N = 404	Rural N = 270	Metropolitan N = 643	Micropolitan N = 117	Small Town N = 55	Rural N = 31
Female (vs. not Female) *	71%	69%	72%	66%	71%	63%	80%	68%
Median Age (Interquartile Interval), Years	74 (69, 81)	74 (69, 81)	74 (69, 80)	74 (69, 82)	81 (73, 87)	80 (74, 85)	81 (72, 88)	79 (70, 87)
Physical Disability *	58%	65%	66%	62%	59%	55%	57%	66%
Unknown	68	9	5	6	27	4	2	2
Developmental Disability *	3.1%	2.7%	3.8%	3.1%	4.9%	6.4%	9.8%	0%
Unknown	107	18	8	9	47	8	4	3
Brain Injury *	7.2%	6.6%	13%	11%	11%	11%	11%	31%
Unknown	97	19	6	4	41	8	2	2
Mental Health Condition	24%	25%	31%	29%	35%	36%	30%	30%
Unknown	56	10	5	2	37	9	2	1
Multiple Chronic Conditions *	19%	22%	29%	24%	73%	69%	67%	81%
Medicare Enrollee *	90%	91%	95%	93%	91%	97%	96%	100%
Unknown	346	60	27	27	78	14	7	3
Marital Status *								
Single	19%	15%	16%	16%	14%	9.1%	11%	0%
Married/Domestic Partner	18%	17%	22%	21%	27%	21%	24%	29%
Separated/Divorced	32%	32%	34%	31%	21%	31%	20%	29%
Widowed	31%	36%	28%	32%	39%	39%	44%	43%
Unknown	256	31	13	4	45	7	1	3
Living Arrangement *								
Alone	58%	65%	64%	65%	27%	41%	36%	30%
Family	38%	33%	33%	32%	61%	48%	57%	63%
Other	3.7%	1.9%	2.9%	2.4%	11%	11%	7.5%	7.4%
Unknown	84	14	25	18	24	3	2	4
Race/Ethnicity *								
Black or African-American	31%	9.6%	7.2%	3.5%	27%	5.2%	9.4%	6.5%
Hispanic or Latino	5.9%	3.7%	9.0%	2.3%	10%	10%	9.4%	3.2%
White	50%	82%	81%	89%	48%	78%	79%	81%
Asian/Multiracial/Other	12%	4.5%	3.3%	5.4%	15%	6.1%	1.9%	9.7%
Unknown	106	16	13	11	36	2	2	0
Overall Health *								
Good	32%	32%	33%	32%	27%	31%	35%	35%
Poor/Fair	58%	58%	50%	53%	64%	56%	56%	52%
Very Good/Excellent	9.8%	9.7%	17%	15%	9.1%	13%	9.3%	13%
Unknown	104	17	21	13	19	5	1	0
Proxy Respondent *	16%	11%	9.4%	7.0%	66%	46%	62%	52%

* *p* < 0.05.

**Table 3:** Service use and consumer-reported unmet service needs among older adult consumers of publicly funded home- and community-based services: The National Core Indicators–Aging & Disability Survey 2022–2023.

	No Dementia				Dementia			
Characteristic	Metropolitan N = 3251	Micropolitan N = 685	Small Town N = 404	Rural N = 270	Metropolitan N = 643	Micropolitan N = 117	Small Town N = 55	Rural N = 31
***Service Use***								
Personal Care *	40%	32%	26%	27%	43%	32%	33%	31%
Homemaker *	20%	23%	32%	36%	14%	20%	22%	26%
Delivered Meal	32%	37%	28%	32%	30%	34%	37%	26%
Adult Day Services *	7.0%	1.8%	1.0%	1.0%	10%	3.2%	1.8%	5.7%
Transportation	5.5%	6.2%	4.2%	6.6%	5.4%	5.0%	4.4%	1.4%
Caregiver Support	1.6%	3.7%	1.2%	<0.1%	4.2%	7.6%	4.6%	2.5%
***Unmet Service Needs***								
Personal Care	10%	9.0%	7.6%	7.4%	12%	14%	9.4%	13%
Homemaker	13%	14%	13%	14%	11%	18%	11%	15%
Delivered Meal	5.4%	6.4%	7.5%	4.3%	3.9%	7.9%	3.2%	4.0%
Adult Day Services	3.1%	1.7%	3.3%	1.9%	3.4%	5.8%	2.5%	1.6%
Transportation	10%	9.3%	9.9%	12%	6.5%	11%	3.4%	11%
Caregiver Support *	1.7%	2.1%	3.3%	1.5%	3.5%	2.6%	4.6%	3.9%

* *p* < 0.05.

**Table 4:** Incremental variance in service use explained by place-based factors among older adult consumers of publicly funded home- and community-based services: The National Core Indicators—Aging & Disability Survey 2022–2023.

Service	Model	Mean AIC	Mean McFadden R^2^	Change in McFadden R^2^ (% Change from Previous Model)
Personal services	1	6559.22	0.244	NA
	2	6436.85	0.262	7.38
	3	6177.83	0.295	12.60
Homemaker services	1	5209.37	0.247	NA
	2	5133.69	0.263	6.48
	3	4801.23	0.315	19.77
Delivered meal services	1	5982.73	0.287	NA
	2	5918.06	0.298	3.83
	3	5628.73	0.336	12.75
Adult day services	1	2227.03	0.235	NA
	2	2134.64	0.279	18.72
	3	2058.82	0.314	12.54
Transportation services	1	2146.25	0.221	NA
	2	2133.19	0.238	7.69
	3	2106.11	0.257	7.98
Caregiver support	1	993.30	0.314	NA
	2	980.62	0.348	10.83
	3	894.06	0.427	22.70

AIC—Akaike Information Criterion; NA—not applicable. Note: Model 1 is adjusted for dementia status, state (location of the participant), and proxy response (vs. self-response). Model 2 is adjusted for Model 1 covariates + person-level attributes including sociodemographic and health-related variables. Model 3 is adjusted for Model 2 covariates + PBFs.

**Table 5:** Incremental variance in consumer-reported unmet service needs explained by place-based factors among older adult consumers of publicly funded home- and community-based services: The National Core Indicators—Aging & Disability Survey 2022–2023.

Service	Model	Mean AIC	McFadden R^2^	Change in McFadden R^2^ (% Change from Previous Model)
Overall unmet need (all HCBS)	1	6719.86	0.061	NA
	2	6613.52	0.081	32.79
	3	6341.08	0.123	51.85
Personal services	1	4060.85	0.050	NA
	2	3988.27	0.075	50.00
	3	3859.31	0.111	48.00
Homemaker services	1	4637.91	0.103	NA
	2	4571.36	0.122	18.45
	3	4368.66	0.166	36.07
Delivered meal services	1	2622.79	0.068	NA
	2	2569.39	0.100	47.06
	3	2465.07	0.146	46.00
Adult day services	1	1619.31	0.083	NA
	2	1620.06	0.103	24.10
	3	1580.33	0.140	35.92
Transportation services	1	3949.89	0.060	NA
	2	3865.32	0.089	48.33
	3	3736.40	0.126	41.57
Caregiver support/respite	1	1227.06	0.130	NA
	2	1210.60	0.167	28.46
	3	1146.41	0.232	38.92

AIC—Akaike Information Criterion; NA—not applicable. Note: Model 1 is adjusted for dementia status, state (location of the participant), and proxy response (vs. self-response). Model 2 is adjusted for Model 1 covariates + person-level attributes including sociodemographic and health-related variables. Model 3 is adjusted for Model 2 covariates + PBFs.

## Data Availability

Restrictions apply to the availability of these data. Data were obtained from the Health Services Research Institute (HSRI) and can be requested from them. The data use agreement between the University of Minnesota and the Health Services Research Institute (HSRI) does not allow sharing of NCI-AD data with the public.

## Supplementary Materials

The following supporting information can be downloaded at https://www.mdpi.com/article/10.3390/ijerph22091461/s1, Table S1: Survey Questions for Service Use and Unmet Needs from National Core Indicators—Aging and Disability Survey; Table S2: Adjusted odds ratios (AOR) and 95% confidence intervals (CIs) for HCBS use: Model 1 with Dementia Status. Models are also adjusted for state ID; Table S3: Adjusted odds ratios and 95% confidence intervals (CIs) for HCBS use: Model 2 with Person-Level Factors. Models are also adjusted for state ID; Table S4: Adjusted odds ratios and 95% confidence intervals (CIs) for HCBS use: Model 3 with Person-Level Plus Place-Based Factors. Models are also adjusted for state ID; Table S5: Adjusted odds ratios (AOR) and 95% confidence intervals (CIs) for Unmet HCBS Needs: Model 1 with Dementia Status. Models are also adjusted for state ID; Table S6: Adjusted odds ratios and 95% confidence intervals (CIs) for Unmet HCBS Needs: Model 2 with Person-Level Factors. Models are also adjusted for state ID; Table S7: Adjusted odds ratios and 95% confidence intervals (CIs) for Unmet HCBS Needs: Model 3 with Person-Level Plus Place-Based Factors. Models are also adjusted for state ID.
