# Supplementary material for: Associations of Place-Based Factors with Service Use and Consumer-Reported Unmet Service Needs Among Older Adults Using Publicly Funded Home- and Community-Based Services in the United States
Source: Int J Environ Res Public Health. 2025 Sep 22;22(9):1461. doi: 10.3390/ijerph22091461 (PMC12469763; doi:10.3390/ijerph22091461)
Supplement: Supplementary file 1 [file ijerph-22-01461-s001.zip › ijerph-3791989-supplementary.pdf]

Supplemental Tables

Supplemental Table S1. Survey Questions for Service Use and Unmet Needs from National Core Indicators – Aging and Disability Survey.

BI-16. What type of paid long-term care supports is the person receiving? [Check all that apply]	Non-HCBS Services:<br><br>__1 Skilled nursing facility/nursing home services<br><br>HCBS Services:<br><br>__2 Round-the-clock services (bundled services by an HCBS provider that has round-the-clock responsibility for the health and welfare of the person – may be provided in a person's home or in a congregate setting such as group home, shared living, assisted living, etc.).<br><br>__3 Home-based services (services that a person receives in his or her home or apartment, when the provider does not have round-the-clock responsibility for the person's health and welfare).<br><br>__3a Personal care services such as attendant care, personal care assistance (assistance with activities of daily living (ADLs), instrumental activities of daily living (IADLs) and/or health-related tasks, NOT including services required to be provided by a licensed home health agency or under the supervision of a licensed nurse or therapist).

	__3b Homemaker services, chore services (performance of light or heavy housekeeping tasks, NOT including assistance with ADLs or other health-related tasks).<br><br>__3c Companion services (supervision and/or social support, NOT including assistance with ADLs or other health-related tasks, or habilitation; may include performance of light housekeeping tasks).<br><br>__3d Other personal care services such as home health aide, etc.<br><br>__4 Day services (services other than supported employment usually provided outside of a person's home during the working day).<br><br>__4a Social adult day services (support services, NOT including health services or habilitation, provided to adults in a fixed site facility during the working day)<br><br>__4b Community integration services (services specifically intended to assist in participating in community activities, NOT including health services or habilitation; may include supports related to community participation that are provided in the person's residence)<br><br>__4c Adult day health

	__4d Other day services such as prevocational services, day habilitation, education services, etc.<br><br>__5 Equipment, medical supplies, technology and modifications such as personal emergency response system<br><br>(PERS), home and/or vehicle accessibility adaptations, assistive technology, specialized medical equipment disposable medical supplies (purchase or rent of material items, devices, or product systems to improve or maintain a person's functional status).<br><br>__6 Nursing services such as private duty nursing, skilled nursing.<br><br>__7 Other health and therapeutic services not identified above such as health monitoring, medication management, occupational or physical therapy (services to support people in improving or maintaining health or functional capacity).<br><br>__8 Other mental health and behavioral services not identified above such as crisis intervention, behavior support, peer specialist, counseling, etc. (services to support people in improving or maintaining mental or behavioral health).

	__9 Non-medical transportation not provided as part of another category such as round-the-clock services or day services. May include transportation to and from other waiver services, transportation to community activities, and/or the purchase of public transit tokens or passes.<br><br>__10 Case management<br><br>__11 Home delivered meals (prepared meals sent to a person's home).<br><br>__12 Employment assistance, supported employment such as job development, ongoing individual or group supported employment, career planning (assistance to help obtain or maintain paid employment or self-employment).<br><br>__13 Caregiver support such as respite and caregiver counseling and/or training (assistance to people who provide ongoing support to the service recipient, when assisting that support person is the primary purpose)<br><br>__14 Other services not listed (FILL IN) ___________________________________<br><br>__50 NONE<br><br>__98 Don't know

In-Person Survey-83. Do the long-term care services you receive meet your current needs and goals?	_N/A – Person said they are not receiving services<br><br>_Yes, completely, all needs and goals<br><br>_Some needs and goals<br><br>_No, not at all, needs or goals are not met<br><br>_Don’t Know<br><br>_Unclear/refused/no response

In-Person Survey-84. What additional long-term care services might help you meet your needs and goals? [Check all that apply	_N/A – Services meet all needs<br><br>_Skilled nursing facility, nursing home services<br><br>_Personal care assistance, personal care services<br><br>_Home maker/chore services<br><br>_Healthcare home services, home health<br><br>_Home delivered meals<br><br>_Adult day services<br><br>_Transportation<br><br>_Respite/family caregiver support<br><br>_Health care<br><br>_Mental health care

	_Dental care<br><br>_Housing assistance<br><br>_Heating/cooling assistance<br><br>_Hospice<br><br>_Funeral Planning<br><br>_Other [fill in]<br><br>_Don’t Know<br><br>_Unclear/refused/no response

Supplemental Table S2. Adjusted odds ratios (AOR) and 95% confidence intervals (CIs) for HCBS use: Model 1 with Dementia Status. Models are also adjusted for state ID.

	Personal Care			Homemaker/Chore			Delivered Meals			Adult Day services			Transportation			Caregiver Support			Overall		
**Characteristic**	**AOR (95% CI)**1	**95% CI**1	**p-value**	**AOR (95% CI)**1	**95% CI**1	**p-value**	**AOR (95% CI)**1	**95% CI**1	**p-value**	**AOR (95% CI)**1	**95% CI**1	**p-value**	**AOR (95% CI)**1	**95% CI**1	**p-value**	**AOR (95% CI)**1	**95% CI**1	**p-value**	**AOR (95% CI)**1	**95% CI**1	**p-value**
ADRD	1.15	0.95, 1.40	0.2	1.06	0.83, 1.35	0.7	1.07	0.86, 1.33	0.6	1.18	0.83, 1.67	0.4	1.56	1.07, 2.26	0.019	2.17	1.35, 3.50	0.001	1.13	0.92, 1.39	0.2
Proxy	1.37	1.16, 1.61	<0.001	0.54	0.44, 0.67	<0.001	0.67	0.56, 0.81	<0.001	1.08	0.81, 1.45	0.6	0.33	0.22, 0.49	<0.001	1.30	0.84, 2.02	0.2	0.92	0.76, 1.10	0.3

[1]CI = Confidence Interval

Supplemental Table S3. Adjusted odds ratios and 95% confidence intervals (CIs) for HCBS use: Model 2 with Person-Level Factors. Models are also adjusted for state ID.

	Personal Care			Homemaker/Chore			Delivered Meals			Adult Day services			Transportation			Caregiver Support			Overall		
**Characteristic**	**AOR (95% CI)**1	**95% CI**1	**p-value**	**AOR (95% CI)**1	**95% CI**1	**p-value**	**AOR (95% CI)**1	**95% CI**1	**p-value**	**AOR (95% CI)**1	**95% CI**1	**p-value**	**AOR (95% CI)**1	**95% CI**1	**p-value**	**AOR (95% CI)**1	**95% CI**1	**p-value**	**AOR (95% CI)**1	**95% CI**1	**p-value**
Female (vs not Female)																					
No	—	—		—	—		—	—		—	—		—	—		—	—		—	—	
Yes	1.24	1.06, 1.45	0.006	1.26	1.07, 1.48	0.007	0.69	0.59, 0.80	<0.001	0.83	0.63, 1.11	0.2	0.87	0.66, 1.15	0.3	0.84	0.54, 1.30	0.4	0.99	0.85, 1.15	>0.9
Race/Ethnicity																					
Asian/Multi/Other	—	—		—	—		—	—		—	—		—	—		—	—		—	—	
Black or African-American	0.97	0.76, 1.23	0.8	0.90	0.65, 1.25	0.5	1.89	1.39, 2.58	<0.001	0.47	0.31, 0.72	<0.001	1.02	0.63, 1.66	>0.9	3.80	1.34, 10.8	0.013	0.78	0.59, 1.04	0.10

Hispanic or Latino	0.69	0.51, 0.94	0.020	0.82	0.51, 1.29	0.4	2.33	1.51, 3.59	<0.001	0.25	0.15, 0.43	<0.001	0.56	0.29, 1.08	0.084	2.39	0.73, 7.83	0.15	0.49	0.35, 0.70	<0.001
White	0.69	0.56, 0.86	<0.001	0.95	0.70, 1.30	0.8	2.35	1.74, 3.18	<0.001	0.30	0.20, 0.44	<0.001	0.88	0.56, 1.38	0.6	3.15	1.10, 9.03	0.033	0.64	0.50, 0.82	<0.001
ADRD	1.11	0.91, 1.35	0.3	1.12	0.87, 1.44	0.4	1.06	0.84, 1.34	0.6	1.19	0.83, 1.70	0.3	1.71	1.16, 2.52	0.007	1.95	1.19, 3.21	0.008	1.09	0.88, 1.35	0.4
Physical Disability	1.37	1.17, 1.60	<0.001	1.03	0.86, 1.23	0.7	0.84	0.72, 0.99	0.034	0.66	0.49, 0.90	0.008	0.95	0.70, 1.28	0.7	0.92	0.59, 1.43	0.7	0.96	0.82, 1.13	0.7
Developmental Disability	0.78	0.53, 1.16	0.2	1.06	0.66, 1.70	0.8	1.21	0.81, 1.80	0.3	1.84	1.03, 3.29	0.041	1.02	0.47, 2.19	>0.9	2.75	1.10, 6.84	0.030	1.27	0.87, 1.85	0.2
Brain Injury	0.83	0.65, 1.07	0.15	0.87	0.64, 1.17	0.3	1.17	0.87, 1.56	0.3	1.24	0.75, 2.05	0.4	1.66	1.05, 2.63	0.029	0.92	0.38, 2.21	0.8	0.92	0.73, 1.16	0.5
Mental Health Condition	0.99	0.84, 1.16	0.9	1.15	0.96, 1.39	0.14	1.11	0.92, 1.33	0.3	1.22	0.90, 1.64	0.2	1.09	0.82, 1.45	0.6	1.43	0.92, 2.23	0.11	1.05	0.88, 1.25	0.6
Overall Health																					
Good	—	—		—	—		—	—		—	—		—	—		—	—		—	—	

Poor/Fair	1.20	1.04, 1.39	0.010	1.05	0.88, 1.25	0.6	0.92	0.79, 1.08	0.3	0.83	0.62, 1.12	0.2	0.93	0.70, 1.23	0.6	0.80	0.51, 1.26	0.3	1.05	0.90, 1.23	0.5
Very Good/Excellent	0.89	0.71, 1.12	0.3	1.05	0.82, 1.34	0.7	0.86	0.68, 1.09	0.2	1.31	0.85, 2.04	0.2	1.48	1.00, 2.19	0.052	0.47	0.20, 1.10	0.081	0.94	0.75, 1.19	0.6
Living Arrangement																					
Alone	—	—		—	—		—	—		—	—		—	—		—	—		—	—	
Family	0.92	0.78, 1.08	0.3	0.55	0.45, 0.67	<0.001	0.98	0.81, 1.17	0.8	1.53	1.10, 2.12	0.011	0.74	0.53, 1.03	0.078	1.46	0.88, 2.40	0.14	0.85	0.72, 1.00	0.051
Other	3.32	2.43, 4.55	<0.001	0.57	0.36, 0.91	0.019	0.59	0.38, 0.92	0.019	1.76	0.97, 3.21	0.064	0.66	0.33, 1.30	0.2	0.56	0.15, 2.01	0.4	3.43	2.40, 4.89	<0.001
Medicare Enrollee	0.80	0.58, 1.10	0.2	2.00	1.31, 3.07	0.002	1.11	0.84, 1.46	0.5	0.89	0.48, 1.65	0.7	1.35	0.70, 2.60	0.4	1.02	0.36, 2.92	>0.9	0.94	0.71, 1.25	0.7
Have Legal Guardian	1.22	0.88, 1.69	0.2	0.92	0.59, 1.44	0.7	0.81	0.58, 1.15	0.2	0.83	0.44, 1.57	0.6	0.58	0.28, 1.19	0.14	1.28	0.63, 2.60	0.5	1.24	0.88, 1.76	0.2
Marital Status																					
Single	—	—		—	—		—	—		—	—		—	—		—	—		—	—	

Married/Domestic Partner	0.97	0.76, 1.23	0.8	0.87	0.65, 1.17	0.4	1.08	0.83, 1.41	0.6	0.49	0.30, 0.78	0.003	0.59	0.37, 0.94	0.026	3.21	1.34, 7.68	0.009	0.88	0.68, 1.15	0.4
Separated/Divorced	0.93	0.76, 1.14	0.5	0.78	0.62, 0.99	0.038	1.07	0.86, 1.32	0.6	0.57	0.37, 0.86	0.008	0.79	0.56, 1.11	0.2	1.76	0.76, 4.06	0.2	0.83	0.67, 1.02	0.077
Widowed	0.94	0.76, 1.16	0.6	0.81	0.64, 1.01	0.066	1.00	0.80, 1.25	>0.9	0.64	0.42, 0.95	0.029	0.69	0.49, 0.99	0.044	2.25	1.02, 4.96	0.045	0.79	0.64, 0.99	0.037
Proxy	1.30	1.09, 1.55	0.004	0.69	0.54, 0.88	0.003	0.70	0.58, 0.85	<0.001	0.92	0.68, 1.26	0.6	0.42	0.28, 0.65	<0.001	1.07	0.66, 1.72	0.8	0.85	0.70, 1.02	0.087

[1]CI = Confidence Interval

Supplemental Table S4. Adjusted odds ratios and 95% confidence intervals (CIs) for HCBS use: Model 3 with Person-Level Plus Place-Based Factors. Models are also adjusted for state ID.

	Personal Care			Homemaker/Chore			Delivered Meals			Adult Day services			Transportation			Caregiver Support			Overall		
**Characteristic**	**AOR (95% CI)**1	**95% CI**1	**p-value**	**AOR (95% CI)**1	**95% CI**1	**p-value**	**AOR (95% CI)**1	**95% CI**1	**p-value**	**AOR (95% CI)**1	**95% CI**1	**p-value**	**AOR (95% CI)**1	**95% CI**1	**p-value**	**AOR (95% CI)**1	**95% CI**1	**p-value**	**AOR (95% CI)**1	**95% CI**1	**p-value**
Female (vs not Female)																					
No	—	—		—	—		—	—		—	—		—	—		—	—		—	—	
Yes	1.26	1.08, 1.48	0.003	1.26	1.06, 1.50	0.009	0.70	0.60, 0.82	<0.001	0.81	0.61, 1.09	0.2	0.88	0.67, 1.17	0.4	0.93	0.59, 1.49	0.8	1.00	0.86, 1.16	>0.9
Race/Ethnicity																					
Asian/Multi/Other	—	—		—	—		—	—		—	—		—	—		—	—		—	—	
Black or African-American	0.95	0.74, 1.24	0.7	0.82	0.58, 1.16	0.3	1.85	1.33, 2.59	<0.001	0.47	0.30, 0.76	0.002	1.06	0.62, 1.82	0.8	2.78	0.93, 8.29	0.067	0.74	0.55, 1.00	0.051

Hispanic or Latino	0.76	0.55, 1.07	0.11	0.84	0.52, 1.37	0.5	2.35	1.49, 3.70	<0.001	0.27	0.15, 0.49	<0.001	0.68	0.33, 1.37	0.3	1.95	0.50, 7.65	0.3	0.55	0.38, 0.80	0.002
White	0.76	0.60, 0.95	0.016	0.95	0.69, 1.31	0.8	2.10	1.53, 2.88	<0.001	0.33	0.22, 0.50	<0.001	0.80	0.50, 1.30	0.4	1.37	0.47, 4.00	0.6	0.67	0.52, 0.86	0.002
ADRD	1.10	0.90, 1.35	0.3	1.13	0.87, 1.46	0.3	1.07	0.84, 1.36	0.6	1.27	0.88, 1.83	0.2	1.72	1.16, 2.56	0.007	2.10	1.23, 3.58	0.007	1.08	0.86, 1.34	0.5
Physical Disability	1.35	1.15, 1.58	<0.001	1.02	0.84, 1.23	0.8	0.82	0.69, 0.97	0.022	0.65	0.48, 0.88	0.006	0.96	0.71, 1.31	0.8	1.00	0.63, 1.59	>0.9	0.96	0.82, 1.13	0.6
Developmental Disability	0.82	0.55, 1.21	0.3	1.08	0.66, 1.77	0.8	1.14	0.75, 1.73	0.5	1.98	1.10, 3.57	0.024	0.98	0.45, 2.11	>0.9	2.36	0.86, 6.47	0.10	1.29	0.88, 1.89	0.2
Brain Injury	0.84	0.65, 1.09	0.2	0.87	0.64, 1.17	0.4	1.12	0.83, 1.52	0.5	1.23	0.74, 2.05	0.4	1.69	1.06, 2.69	0.028	1.10	0.45, 2.69	0.8	0.92	0.72, 1.18	0.5
Mental Health Condition	0.98	0.83, 1.16	0.8	1.15	0.95, 1.39	0.14	1.08	0.90, 1.31	0.4	1.29	0.95, 1.74	0.10	1.07	0.80, 1.43	0.7	1.11	0.70, 1.78	0.7	1.04	0.87, 1.25	0.6
ZIP Code RUCA Classification																					
Metropolitan	—	—		—	—		—	—		—	—		—	—		—	—		—	—	

Micropolitan	0.89	0.72, 1.11	0.3	1.00	0.79, 1.27	>0.9	0.96	0.77, 1.20	0.7	0.44	0.25, 0.79	0.006	0.82	0.55, 1.24	0.4	2.40	1.31, 4.41	0.005	0.93	0.75, 1.15	0.5
Rural	0.71	0.50, 1.01	0.059	1.67	1.18, 2.38	0.004	1.21	0.84, 1.74	0.3	0.43	0.16, 1.15	0.093	0.72	0.40, 1.31	0.3	0.18	0.02, 1.53	0.12	1.00	0.70, 1.43	>0.9
Small town	0.73	0.55, 0.97	0.031	1.52	1.14, 2.04	0.005	0.84	0.63, 1.12	0.2	0.29	0.11, 0.74	0.010	0.61	0.36, 1.05	0.075	0.97	0.38, 2.50	>0.9	0.84	0.64, 1.09	0.2
Overall Health																					
Good	—	—		—	—		—	—		—	—		—	—		—	—		—	—	
Poor/Fair	1.22	1.05, 1.41	0.007	1.07	0.90, 1.28	0.4	0.94	0.80, 1.10	0.4	0.80	0.59, 1.08	0.2	0.93	0.70, 1.23	0.6	0.83	0.51, 1.35	0.5	1.07	0.92, 1.25	0.4
Very Good/Excellent	0.90	0.71, 1.14	0.4	1.02	0.78, 1.32	>0.9	0.84	0.65, 1.07	0.2	1.34	0.85, 2.09	0.2	1.47	0.98, 2.19	0.061	0.52	0.22, 1.24	0.14	0.92	0.73, 1.17	0.5
Living Arrangement																					
Alone	—	—		—	—		—	—		—	—		—	—		—	—		—	—	
Family	0.94	0.79, 1.11	0.5	0.56	0.45, 0.68	<0.001	0.95	0.79, 1.14	0.6	1.50	1.08, 2.10	0.016	0.74	0.53, 1.03	0.076	1.36	0.80, 2.31	0.3	0.85	0.71, 1.00	0.052

Other	3.55	2.58, 4.86	<0.001	0.58	0.36, 0.92	0.021	0.61	0.39, 0.94	0.027	1.68	0.91, 3.10	0.10	0.67	0.34, 1.34	0.3	0.63	0.17, 2.34	0.5	3.67	2.53, 5.31	<0.001
Medicare Enrollee	0.81	0.59, 1.12	0.2	1.99	1.29, 3.07	0.002	1.06	0.80, 1.40	0.7	0.88	0.47, 1.64	0.7	1.30	0.67, 2.53	0.4	0.77	0.25, 2.41	0.7	0.94	0.71, 1.25	0.7
Have Legal Guardian	1.18	0.85, 1.65	0.3	0.92	0.58, 1.46	0.7	0.82	0.58, 1.15	0.2	0.86	0.46, 1.61	0.6	0.59	0.29, 1.21	0.15	1.41	0.67, 2.96	0.4	1.22	0.85, 1.75	0.3
Marital Status																					
Single	—	—		—	—		—	—		—	—		—	—		—	—		—	—	
Married/Domestic Partner	0.99	0.77, 1.26	>0.9	0.88	0.65, 1.17	0.4	1.14	0.86, 1.50	0.4	0.47	0.29, 0.77	0.003	0.59	0.36, 0.96	0.035	3.25	1.30, 8.14	0.012	0.92	0.70, 1.20	0.5
Separated/Divorced	0.94	0.76, 1.15	0.5	0.78	0.61, 0.99	0.042	1.07	0.86, 1.33	0.5	0.57	0.37, 0.88	0.011	0.78	0.55, 1.11	0.2	1.50	0.60, 3.71	0.4	0.84	0.68, 1.04	0.11
Widowed	0.94	0.76, 1.17	0.6	0.81	0.64, 1.03	0.082	1.02	0.81, 1.29	0.8	0.62	0.41, 0.95	0.027	0.69	0.48, 0.99	0.046	2.06	0.89, 4.76	0.092	0.82	0.66, 1.02	0.080
% white	1.00	0.96, 1.05	0.9	0.94	0.90, 0.99	0.020	1.04	1.00, 1.09	0.062	0.97	0.89, 1.05	0.4	1.08	0.99, 1.18	0.090	1.06	0.93, 1.21	0.4	1.00	0.95, 1.05	0.9

% high needs (10% increase)	0.80	0.67, 0.95	0.011	0.93	0.77, 1.13	0.5	0.93	0.78, 1.12	0.5	1.40	0.98, 1.98	0.064	0.99	0.69, 1.41	>0.9	0.92	0.51, 1.67	0.8	0.86	0.73, 1.02	0.077
% hispanic (10% increase)	0.98	0.90, 1.07	0.7	0.94	0.84, 1.05	0.3	0.99	0.88, 1.11	0.9	0.95	0.81, 1.11	0.5	0.87	0.73, 1.04	0.12	1.37	0.92, 2.05	0.12	0.94	0.86, 1.03	0.2
% foreign born (10% increase)	1.03	0.91, 1.15	0.7	1.00	0.86, 1.17	>0.9	0.80	0.69, 0.92	0.002	1.00	0.83, 1.21	>0.9	0.91	0.72, 1.16	0.5	0.29	0.16, 0.50	<0.001	1.02	0.89, 1.16	0.8
% dropout (10% increase)	0.83	0.69, 0.99	0.035	1.12	0.92, 1.36	0.3	1.16	0.97, 1.39	0.11	1.07	0.74, 1.55	0.7	1.07	0.75, 1.53	0.7	1.33	0.69, 2.56	0.4	1.08	0.91, 1.30	0.4
% crowding (10% increase)	1.14	0.76, 1.71	0.5	0.60	0.36, 0.99	0.045	0.97	0.62, 1.52	0.9	1.21	0.58, 2.52	0.6	1.26	0.62, 2.59	0.5	1.16	0.24, 5.66	0.9	0.69	0.45, 1.05	0.082
broadband (10% increase)	0.87	0.77, 0.98	0.020	1.11	0.97, 1.27	0.14	0.95	0.83, 1.07	0.4	1.21	0.95, 1.56	0.13	0.89	0.70, 1.13	0.3	1.23	0.83, 1.83	0.3	0.98	0.87, 1.10	0.8
# elderly/disabled person services per sq mile w/ sales>0	1.03	0.90, 1.19	0.6	0.90	0.71, 1.14	0.4	1.10	0.92, 1.31	0.3	1.16	0.93, 1.45	0.2	0.86	0.61, 1.21	0.4	0.26	0.08, 0.82	0.022	1.14	0.98, 1.32	0.082
# elderly/disabled person services	1.02	0.99, 1.05	0.3	1.03	0.99, 1.07	0.15	0.99	0.96, 1.03	0.7	0.95	0.89, 1.02	0.13	1.13	1.07, 1.19	<0.001	1.04	0.93, 1.16	0.5	1.01	0.97, 1.04	0.6

Proxy	1.30	1.08, 1.56	0.005	0.69	0.54, 0.89	0.004	0.69	0.56, 0.84	<0.001	0.84	0.61, 1.16	0.3	0.44	0.29, 0.68	<0.001	1.23	0.73, 2.07	0.4	0.86	0.71, 1.05	0.14

1CI = Confidence Interval

Supplemental Table S5. Adjusted odds ratios (AOR) and 95% confidence intervals (CIs) for Unmet HCBS Needs: Model 1 with Dementia Status. Models are also adjusted for state ID.

	**Personal Care**			**Homemaker/Chore**			**Delivered Meals**			**Adult Day services**			**Transportation**			**Caregiver Support**			**Overall**		
**Characteristic**	AOR (95% CI)1	95% CI1	p-value	AOR (95% CI)1	95% CI1	p-value	AOR (95% CI)1	95% CI1	p-value	AOR (95% CI)1	95% CI1	p-value	AOR (95% CI)1	95% CI1	p-value	AOR (95% CI)1	95% CI1	p-value	AOR (95% CI)1	95% CI1	p-value
ADRD	1.13	0.83, 1.55	0.4	1.09	0.81, 1.48	0.6	1.03	0.61, 1.76	0.9	1.41	0.85, 2.36	0.2	0.94	0.66, 1.34	0.7	1.18	0.64, 2.16	0.6	1.04	0.83, 1.30	0.7
Proxy	1.20	0.91, 1.57	0.2	0.65	0.50, 0.86	0.003	0.76	0.49, 1.18	0.2	0.99	0.59, 1.64	>0.9	0.48	0.32, 0.70	<0.001	4.02	2.31, 6.98	<0.001			
Actual Service	0.76	0.57, 1.01	0.058	0.72	0.56, 0.91	0.007	0.97	0.68, 1.39	0.9	0.76	0.30, 1.94	0.6	1.18	0.74, 1.90	0.5	0.69	0.12, 3.82	0.7	0.76	0.62, 0.94	0.010

[1]CI = Confidence Interval

Supplemental Table S6. Adjusted odds ratios and 95% confidence intervals (CIs) for Unmet HCBS Needs: Model 2 with Person-Level Factors. Models are also adjusted for state ID.

	**Personal Care**			**Homemaker/Chore**			**Delivered Meals**			**Adult Day services**			**Transportation**			**Caregiver Support**			**Overall**		
**Characteristic**	AOR (95% CI)1	95% CI1	p-value	AOR (95% CI)1	95% CI1	p-value	AOR (95% CI)1	95% CI1	p-value	AOR (95% CI)1	95% CI1	p-value	AOR (95% CI)1	95% CI1	p-value	AOR (95% CI)1	95% CI1	p-value	AOR (95% CI)1	95% CI1	p-value
Female (vs not Female)																					
No	—	—		—	—		—	—		—	—		—	—		—	—		—	—	
Yes	0.94	0.73, 1.21	0.6	0.88	0.72, 1.09	0.2	1.02	0.73, 1.41	>0.9	0.74	0.49, 1.10	0.13	0.83	0.67, 1.04	0.10	1.09	0.62, 1.93	0.8	0.93	0.80, 1.09	0.4
Race/Ethnicity																					
Asian/Multi/Other	—	—		—	—		—	—		—	—		—	—		—	—		—	—	
Black or African-American	0.99	0.66, 1.48	>0.9	1.10	0.72, 1.68	0.7	1.75	0.83, 3.67	0.14	2.08	0.83, 5.22	0.12	0.94	0.61, 1.47	0.8	3.51	0.79, 15.5	0.10	1.23	0.90, 1.68	0.2

Hispanic or Latino	0.79	0.46, 1.35	0.4	0.86	0.51, 1.45	0.6	1.90	0.82, 4.38	0.13	0.85	0.28, 2.57	0.8	0.56	0.26, 1.20	0.13	3.60	0.65, 20.0	0.14	0.86	0.56, 1.31	0.5
White	0.77	0.53, 1.12	0.2	0.83	0.57, 1.21	0.3	1.43	0.74, 2.78	0.3	1.40	0.57, 3.40	0.5	1.08	0.74, 1.56	0.7	3.78	0.89, 16.0	0.071	1.10	0.85, 1.43	0.5
ADRD	1.21	0.88, 1.66	0.2	1.10	0.81, 1.51	0.5	1.10	0.63, 1.92	0.7	1.34	0.78, 2.28	0.3	0.99	0.68, 1.43	>0.9	1.14	0.61, 2.13	0.7	1.05	0.84, 1.33	0.7
Physical Disability	1.27	0.99, 1.62	0.061	1.06	0.84, 1.34	0.6	1.00	0.73, 1.36	>0.9	0.90	0.57, 1.43	0.7	0.96	0.74, 1.25	0.8	0.99	0.56, 1.77	>0.9	1.03	0.87, 1.22	0.8
Developmental Disability	1.32	0.78, 2.25	0.3	0.91	0.49, 1.70	0.8	1.20	0.51, 2.79	0.7	0.79	0.29, 2.14	0.6	0.86	0.45, 1.66	0.7	0.65	0.13, 3.37	0.6	0.91	0.60, 1.39	0.7
Brain Injury	1.35	0.97, 1.88	0.080	1.18	0.85, 1.65	0.3	0.90	0.54, 1.50	0.7	1.17	0.63, 2.14	0.6	1.23	0.84, 1.79	0.3	1.30	0.67, 2.55	0.4	1.32	1.05, 1.67	0.019
Mental Health Condition	0.87	0.66, 1.13	0.3	1.26	0.99, 1.60	0.062	1.04	0.72, 1.50	0.8	1.59	1.03, 2.44	0.034	1.32	1.01, 1.72	0.040	0.88	0.47, 1.63	0.7	1.25	1.05, 1.49	0.012
Overall Health																					
Good	—	—		—	—		—	—		—	—		—	—		—	—		—	—	

Poor/Fair	1.64	1.28, 2.12	<0.001	1.70	1.35, 2.14	<0.001	2.11	1.46, 3.06	<0.001	1.17	0.77, 1.77	0.5	1.70	1.32, 2.18	<0.001	1.39	0.79, 2.46	0.3	1.63	1.39, 1.92	<0.001
Very Good/Excellent	0.72	0.46, 1.12	0.14	0.97	0.69, 1.37	0.9	0.96	0.51, 1.81	>0.9	0.95	0.46, 1.94	0.9	0.79	0.49, 1.26	0.3	1.07	0.44, 2.59	0.9	0.88	0.68, 1.15	0.4
Living Arrangement																					
Alone	—	—		—	—		—	—		—	—		—	—		—	—		—	—	
Family	0.75	0.57, 0.99	0.040	0.77	0.59, 1.00	0.048	0.92	0.62, 1.37	0.7	1.11	0.70, 1.75	0.7	0.74	0.54, 0.99	0.045	1.36	0.74, 2.50	0.3	0.79	0.65, 0.96	0.017
Other	0.53	0.28, 1.00	0.051	0.47	0.23, 0.96	0.039	0.49	0.16, 1.50	0.2	0.96	0.41, 2.29	>0.9	0.42	0.21, 0.85	0.016	0.72	0.13, 4.17	0.7	0.57	0.38, 0.88	0.010
Medicare Enrollee	0.94	0.59, 1.50	0.8	0.71	0.47, 1.07	0.10	0.51	0.28, 0.91	0.025	0.81	0.31, 2.17	0.7	1.28	0.84, 1.94	0.3	0.49	0.18, 1.32	0.2	0.91	0.69, 1.20	0.5
Have Legal Guardian	1.04	0.69, 1.58	0.8	1.09	0.66, 1.80	0.7	0.59	0.21, 1.71	0.3	1.07	0.44, 2.60	0.9	0.78	0.44, 1.40	0.4	0.94	0.26, 3.40	>0.9	0.98	0.69, 1.37	0.9
Marital Status																					
Single	—	—		—	—		—	—		—	—		—	—		—	—		—	—	

Married/Domestic Partner	1.16	0.79, 1.69	0.4	1.29	0.89, 1.87	0.2	1.24	0.69, 2.24	0.5	1.00	0.47, 2.16	>0.9	0.86	0.57, 1.30	0.5	2.12	0.86, 5.23	0.10	1.19	0.92, 1.53	0.2
Separated/Divorced	1.31	0.98, 1.74	0.063	1.21	0.89, 1.63	0.2	1.29	0.81, 2.06	0.3	1.28	0.70, 2.35	0.4	1.10	0.81, 1.48	0.5	1.20	0.46, 3.10	0.7	1.27	1.02, 1.57	0.031
Widowed	0.87	0.61, 1.23	0.4	1.09	0.80, 1.48	0.6	0.93	0.58, 1.49	0.8	1.22	0.64, 2.33	0.5	0.75	0.55, 1.04	0.083	1.27	0.49, 3.28	0.6	0.99	0.78, 1.25	>0.9
Proxy	1.28	0.96, 1.70	0.094	0.67	0.50, 0.89	0.007	0.77	0.48, 1.22	0.3	1.00	0.58, 1.73	>0.9	0.56	0.37, 0.86	0.008	3.41	1.92, 6.04	<0.001			
Actual Service	0.73	0.54, 0.99	0.041	0.71	0.55, 0.91	0.007	0.95	0.66, 1.37	0.8	0.75	0.29, 1.92	0.5	1.09	0.67, 1.77	0.7	0.56	0.10, 3.33	0.5	0.83	0.66, 1.03	0.10

[1]CI = Confidence Interval

Supplemental Table S7. Adjusted odds ratios and 95% confidence intervals (CIs) for Unmet HCBS Needs: Model 3 with Person-Level Plus Place-Based Factors. Models are also adjusted for state ID.

	**Personal Care**			**Homemaker/Chore**			**Delivered Meals**			**Adult Day services**			**Transportation**			**Caregiver Support**			**Overall**		
**Characteristic**	AOR (95% CI)1	95% CI1	p-value	AOR (95% CI)1	95% CI1	p-value	AOR (95% CI)1	95% CI1	p-value	AOR (95% CI)1	95% CI1	p-value	AOR (95% CI)1	95% CI1	p-value	AOR (95% CI)1	95% CI1	p-value	AOR (95% CI)1	95% CI1	p-value
Female (vs not Female)																					
No	—	—		—	—		—	—		—	—		—	—		—	—		—	—	
Yes	0.91	0.71, 1.18	0.5	0.86	0.70, 1.06	0.2	0.99	0.71, 1.38	>0.9	0.75	0.50, 1.13	0.2	0.84	0.66, 1.05	0.13	1.09	0.61, 1.93	0.8	0.91	0.77, 1.07	0.2
Race/Ethnicity																					
Asian/Multi/Other	—	—		—	—		—	—		—	—		—	—		—	—		—	—	
Black or African-American	1.11	0.70, 1.74	0.7	1.23	0.79, 1.93	0.4	1.64	0.77, 3.49	0.2	1.96	0.77, 5.03	0.2	0.94	0.59, 1.51	0.8	5.13	0.80, 32.9	0.084	1.32	0.95, 1.83	0.10

Hispanic or Latino	0.85	0.48, 1.50	0.6	0.87	0.49, 1.52	0.6	1.66	0.68, 4.03	0.3	0.84	0.27, 2.56	0.8	0.58	0.26, 1.25	0.2	4.80	0.57, 40.2	0.15	0.88	0.58, 1.36	0.6
White	0.81	0.55, 1.21	0.3	0.83	0.55, 1.25	0.4	1.41	0.71, 2.79	0.3	1.39	0.55, 3.52	0.5	1.13	0.77, 1.66	0.5	4.87	0.77, 30.7	0.091	1.11	0.85, 1.47	0.4
ADRD	1.23	0.88, 1.70	0.2	1.11	0.81, 1.52	0.5	1.04	0.59, 1.85	0.9	1.33	0.77, 2.30	0.3	0.99	0.67, 1.44	>0.9	1.20	0.62, 2.29	0.6	1.05	0.83, 1.32	0.7
Physical Disability	1.30	1.00, 1.68	0.048	1.07	0.83, 1.36	0.6	1.01	0.73, 1.40	>0.9	0.93	0.59, 1.48	0.8	0.95	0.73, 1.23	0.7	1.03	0.56, 1.92	>0.9	1.03	0.86, 1.23	0.8
Developmental Disability	1.26	0.73, 2.17	0.4	0.84	0.44, 1.58	0.6	1.18	0.48, 2.91	0.7	0.78	0.28, 2.20	0.6	0.83	0.42, 1.63	0.6	0.69	0.13, 3.81	0.7	0.86	0.55, 1.33	0.5
Brain Injury	1.30	0.92, 1.82	0.14	1.12	0.79, 1.59	0.5	0.90	0.53, 1.53	0.7	1.16	0.64, 2.09	0.6	1.21	0.84, 1.76	0.3	1.37	0.69, 2.72	0.4	1.27	1.00, 1.62	0.051
Mental Health Condition	0.87	0.66, 1.15	0.3	1.25	0.98, 1.58	0.074	1.05	0.73, 1.51	0.8	1.60	1.02, 2.51	0.042	1.33	1.01, 1.75	0.040	0.86	0.46, 1.60	0.6	1.25	1.05, 1.50	0.013
ZIP Code RUCA Classification																					
Metropolitan	—	—		—	—		—	—		—	—		—	—		—	—		—	—	

Micropolitan	0.99	0.71, 1.38	>0.9	1.13	0.82, 1.57	0.5	1.27	0.80, 2.01	0.3	0.68	0.32, 1.42	0.3	0.80	0.58, 1.10	0.2	1.08	0.50, 2.34	0.8	0.90	0.71, 1.15	0.4
Rural	0.94	0.53, 1.69	0.8	1.20	0.75, 1.93	0.4	0.91	0.38, 2.16	0.8	0.48	0.14, 1.72	0.3	0.89	0.54, 1.49	0.7	0.89	0.23, 3.50	0.9	0.98	0.68, 1.42	>0.9
Small town	0.81	0.50, 1.31	0.4	0.88	0.59, 1.32	0.5	1.34	0.78, 2.32	0.3	1.01	0.48, 2.13	>0.9	0.81	0.53, 1.23	0.3	1.87	0.85, 4.10	0.12	0.88	0.65, 1.20	0.4
Overall Health																					
Good	—	—		—	—		—	—		—	—		—	—		—	—		—	—	
Poor/Fair	1.66	1.28, 2.14	<0.001	1.67	1.33, 2.11	<0.001	2.10	1.45, 3.03	<0.001	1.14	0.74, 1.74	0.6	1.67	1.29, 2.15	<0.001	1.37	0.77, 2.43	0.3	1.61	1.36, 1.89	<0.001
Very Good/Excellent	0.76	0.48, 1.18	0.2	1.00	0.71, 1.41	>0.9	0.96	0.50, 1.83	0.9	0.95	0.45, 2.00	0.9	0.80	0.50, 1.28	0.4	1.08	0.40, 2.87	0.9	0.90	0.69, 1.17	0.4
Living Arrangement																					
Alone	—	—		—	—		—	—		—	—		—	—		—	—		—	—	
Family	0.75	0.56, 1.00	0.049	0.76	0.58, 0.98	0.038	0.89	0.60, 1.33	0.6	1.10	0.68, 1.77	0.7	0.72	0.53, 0.97	0.030	1.38	0.71, 2.68	0.3	0.77	0.63, 0.94	0.010

Other	0.54	0.29, 1.02	0.058	0.48	0.23, 0.99	0.048	0.47	0.15, 1.43	0.2	1.03	0.43, 2.45	>0.9	0.42	0.20, 0.87	0.019	0.70	0.12, 4.17	0.7	0.58	0.38, 0.90	0.014
Medicare Enrollee	0.97	0.61, 1.53	0.9	0.72	0.48, 1.08	0.11	0.51	0.27, 0.93	0.029	0.81	0.31, 2.14	0.7	1.30	0.84, 2.02	0.2	0.49	0.18, 1.35	0.2	0.93	0.71, 1.23	0.6
Have Legal Guardian	1.03	0.67, 1.57	>0.9	1.10	0.65, 1.86	0.7	0.53	0.17, 1.71	0.3	1.02	0.41, 2.54	>0.9	0.77	0.43, 1.40	0.4	0.92	0.26, 3.25	0.9	0.96	0.67, 1.37	0.8
Marital Status																					
Single	—	—		—	—		—	—		—	—		—	—		—	—		—	—	
Married/Domestic Partner	1.16	0.78, 1.70	0.5	1.28	0.88, 1.86	0.2	1.26	0.68, 2.33	0.5	1.00	0.46, 2.16	>0.9	0.90	0.60, 1.36	0.6	2.08	0.81, 5.33	0.13	1.20	0.92, 1.55	0.2
Separated/Divorced	1.30	0.97, 1.75	0.082	1.20	0.88, 1.62	0.2	1.27	0.79, 2.04	0.3	1.28	0.70, 2.36	0.4	1.11	0.83, 1.49	0.5	1.14	0.42, 3.06	0.8	1.26	1.01, 1.57	0.037
Widowed	0.87	0.62, 1.23	0.4	1.08	0.79, 1.48	0.6	0.94	0.58, 1.51	0.8	1.24	0.65, 2.40	0.5	0.77	0.56, 1.06	0.11	1.24	0.47, 3.31	0.7	0.99	0.79, 1.25	>0.9
% white (10% increase)	1.02	0.95, 1.10	0.5	1.03	0.96, 1.09	0.4	0.96	0.87, 1.06	0.4	1.01	0.89, 1.16	0.8	1.00	0.93, 1.07	>0.9	1.01	0.86, 1.19	>0.9	1.03	0.98, 1.08	0.3

% high needs (10% increase)	0.95	0.74, 1.21	0.7	1.01	0.79, 1.28	>0.9	0.93	0.67, 1.29	0.7	0.92	0.60, 1.39	0.7	0.87	0.67, 1.13	0.3	1.06	0.64, 1.74	0.8	0.91	0.76, 1.09	0.3
% hispanic (10% increase)	1.03	0.89, 1.20	0.7	1.04	0.91, 1.20	0.5	1.08	0.88, 1.32	0.5	1.06	0.78, 1.43	0.7	1.03	0.87, 1.21	0.7	0.87	0.60, 1.25	0.4	1.01	0.91, 1.13	0.8
% foreign born (10% increase)	1.06	0.87, 1.29	0.6	0.93	0.76, 1.13	0.4	0.97	0.73, 1.30	0.9	0.82	0.51, 1.31	0.4	0.94	0.75, 1.19	0.6	0.88	0.53, 1.46	0.6	0.96	0.83, 1.10	0.5
% dropout (10% increase)	0.89	0.68, 1.16	0.4	1.09	0.84, 1.40	0.5	0.87	0.58, 1.31	0.5	0.69	0.41, 1.16	0.2	0.87	0.66, 1.16	0.3	1.88	1.05, 3.38	0.035	0.99	0.81, 1.22	>0.9
% crowding (10% increase)	0.87	0.44, 1.70	0.7	0.98	0.53, 1.83	>0.9	0.92	0.35, 2.43	0.9	1.92	0.55, 6.72	0.3	1.01	0.51, 2.00	>0.9	0.49	0.08, 3.17	0.5	1.02	0.63, 1.65	>0.9
broadband (10% increase)	0.95	0.77, 1.16	0.6	1.09	0.92, 1.30	0.3	0.93	0.71, 1.22	0.6	0.84	0.59, 1.20	0.3	0.87	0.72, 1.07	0.2	1.71	1.01, 2.90	0.045	0.96	0.85, 1.10	0.6
# elderly/disabled person services per sq mile w/ sales>0	1.02	0.81, 1.28	0.9	1.00	0.78, 1.30	>0.9	0.65	0.40, 1.04	0.073	1.03	0.70, 1.53	0.9	0.83	0.61, 1.12	0.2	1.28	0.76, 2.13	0.3	0.92	0.76, 1.12	0.4
# elderly/disabled person services	1.00	0.95, 1.06	>0.9	1.03	0.98, 1.08	0.2	1.08	1.00, 1.16	0.059	1.08	0.98, 1.18	0.11	1.03	0.97, 1.09	0.4	0.96	0.85, 1.09	0.5	1.00	0.97, 1.04	0.8

Proxy	1.25	0.93, 1.66	0.13	0.66	0.50, 0.88	0.005	0.79	0.49, 1.25	0.3	1.01	0.59, 1.73	>0.9	0.57	0.38, 0.86	0.007	3.44	1.89, 6.25	<0.001			
Actual Service	0.74	0.54, 1.00	0.049	0.73	0.57, 0.94	0.015	0.96	0.65, 1.41	0.8	0.75	0.29, 1.95	0.6	1.07	0.66, 1.74	0.8	0.53	0.09, 3.12	0.5	0.82	0.65, 1.03	0.089

[1]CI = Confidence Interval
